# Fermentation improves flavors, bioactive substances, and antioxidant capacity of Bian-Que Triple-Bean Soup by lactic acid bacteria

**DOI:** 10.3389/fmicb.2023.1152654

**Published:** 2023-07-18

**Authors:** Yiming Li, Huixin Yang, Bin Yu, Jiayao Wang, Manli Zhu, Jiao Liu, Zhenjie Zheng, Zhenning Qian, Linya Wei, Huanyong Lv, Lili Zhang, Yunhe Xu

**Affiliations:** ^1^College of Food and Health, Jinzhou Medical University, Jinzhou, China; ^2^Comparative Molecular Biosciences Graduate Program, University of Minnesota – Twin Cities, St. Paul, MN, United States; ^3^Department of Food Science and Engineering, Qilu University of Technology, Jinan, Shandong, China

**Keywords:** Bian-Que Triple-Bean Soup, fermentation, flavor, bioactive substances, differential metabolites analysis

## Abstract

The ancient traditional Chinese drink Bian-Que Triple-Bean Soup made by fermentation (FTBS) of *Lactococcus lactis* subsp*. lactis* YM313 and *Lacticaseibacillus casei* YQ336 is a potential functional drink. The effect of fermentation on the flavor and biological activity of FTBS was evaluated by analyzing its chemical composition. Five volatile flavors were detected in modified FTBS. Fermentation decreased the proportion of nonanal (beany flavor substances) but significantly increased the total flavone contents, phenol contents and many bioactive small molecule substances in FTBS. The changes of these substances led to the significant improvement of FTBS sensory evaluation, antioxidant activity and prebiotic potential. This research provides a theoretical basis for the application of Lactic acid bacteria (LAB) in the fermentation of edible plant-based foods and transformation from traditional food to industrial production.

## 1. Introduction

At present, 88% of deaths in China are caused by chronic diseases, including low immunity, diabetes, cancer, etc. Most of the chronic diseases can be attenuated and even reversed by following a plant-based diet. Traditional Chinese medicine often uses some prescriptions for medicine and food to prevent chronic diseases and achieve good results.

Bian-Que Triple-Bean Soup (TBS) is a traditional Chinese prescription introduced by Bian-Que in the Spring and Autumn Period (BC 770 to BC 476) of ancient China. According to the record of *Huang Di Nei Jing*, TBS is a soup with adzuki bean, mung bean and black soybean as main raw materials that has been used to treat diabetes, anti-inflammatory and improve the overall immunity of consumers (Song et al., 2013). Modern science proves they are rich in digestible proteins, amino acids, carbohydrates and other nutrients. In addition, Adzuki beans are rich in many bioactive substances that have potential biological activities (Song et al., 2013; Kawakami et al., 2018). Mukai and Sato showed that adzuki beans have good antioxidant and anti-inflammatory activities (Mukai and Sato, 2011). Mung beans are rich in phenolic substances (Gan et al., 2017). Luo et al. (2016) showed that phytochemicals, such as vitexin and isovitexin in mung beans provide it antioxidant, anti-inflammatory, and anti-diabetic effects. Black soybeans are rich in polyphenols, isoflavones and saponins that have outstanding antioxidant, anti-hypertensive, antitumor, anticancer, lipid-lowering, anti-inflammatory, and other health benefits (Kim et al., 2019; Tsui and Yang, 2021). Moreover, the three kinds of beans can synergize with each other improving the overall health benefits of TBS (Song et al., 2013). That make TBS beneficial as an antioxidant against free radicals, antibacterial, anti-inflammatory and in the treatment of diabetes, hand, foot, and mouth diseases (Song et al., 2013; Hui, 2020).

The “beany odor,” also called beany flavor, is a long-standing problem in soybean products. Some studies have shown that short chain aliphatic alcohols, ketones, and aldehydes cause this kind of flavor, and aldehydes (such as hexanal, nonanal and cis-6-nonenal) having the greatest impact on the beany taste (Wu et al., 2018; Tao et al., 2022; Wang Z. et al., 2022). LAB fermentation has been used to improve the shelf life and flavor of food. Blagden and Gilliland found that the unsaturated fatty acids in soymilk are degraded into small molecules of alcohol, acid and other flavor substances, and beany odor can be masked by fermented flavors produced during LAB fermentation (Blagden and Gilliland, 2005). Other methods to reduce the beany odor include enzyme treatment, supercritical CO_2_ extraction, new cultivars breeding, genetic engineering, etc. However, some of these methods have hidden risks for food safety and quality compared to LAB fermentation, while the high cost and extra energy consumption also bring about new problems (Tao et al., 2022). Also, LAB fermentation significantly increases the content of bioactive substances improving the prebiotic and antioxidant properties of legume-based beverages. Kim et al. (2019) showed that LAB fermented soymilk of Yak-Kong soybeans has a significantly higher antioxidant capacity. Similarly, Tsui and Yang (2021) found that the black soybeans milk contents of myricetin and isoflavone aglycones increased by 1.61 and 1.95 times, respectively, after the *L. plantarum* fermentation. A study showed that mung bean milk and soymilk produced by *Lactobacillus plantarum* WCFS1 fermentation showed significant improvement in antioxidant properties and the content of total phenolics (Gan et al., 2017). There is a habit of eating fermented bean products in Chinese history. For example, the Beijing Douzhir (mung bean acid slurry) is a drink made from fermented mung beans by LAB, which is known to help combat the high summer temperatures.

Currently, there is little scientific research on TBS, and the active ingredients that play a role in the benefits are not clear, with sufficient modern scientific evidence still lacking with respect to efficacy and safety. Moreover, there are only a few studies optimizing the active ingredients and prebiotic mechanisms of TBS. From the perspective of product processing, TBS is a manually produced home product with undesirable characteristics such as beany odor often associated with soy, which all limit the industrial production and promotion of this product. Accordingly, a strain of *Lactococcus lactis* subsp*. lactis* YM313 screened from Beijing Douzhir which had excellent fermentation flavor (Xu et al., 2018) and a strain of *Lacticaseibacillus casei* YQ336 screened from soybean acid slurry which could rapidly produce acid (Xu et al., 2019) were used as a starter culture for TBS fermentation. The volatile flavor substances changes in TBS and Bian-Que Triple-Bean Soup after fermentation (FTBS) were analyzed by SPME-GC–MS and electronic nose. Meanwhile, the metabolites changes in TBS and FTBS were studied by non-targeted ultra-high performance liquid chromatography-tandem Fourier transform mass spectrometry (UHPLC-QExactiveHF-X). The purpose of this study is to reduce the beany odor, improve the flavor and increase the content of active substances in TBS through LAB fermentation. It provides new ideas and technical references for the development of fermented bean food and medical-food homologous food.

## 2. Materials and methods

### 2.1. Samples and chemicals

Mung bean, adzuki bean, and black soybean were produced in Liaoning province, China. Jasmine green tea was provided by China Tea Co., Ltd. (Hunan, China). *Lacticaseibacillus casei* YQ336 was obtained from the China General Microbiological Culture Collection Centre (CGMCC NO.12796, GenBank KY885466). *Lactococcus lactis* subsp*. lactis* YM313 was obtained from the Guangdong Microbial Culture Collection Centre (GDMCC NO.61831, GenBank OM189539). Standards and lactic, tartaric, acetic, oxalic, and citric acids were provided by Tmrm Quality Inspection Technology Co., Ltd. (Beijing, China).

TBS was prepared according to traditional methods: Firstly, an equal mixture of beans (mung, adzuki and black soybeans 100 g respectively) was added to boiling water (1:8 w/v), with 6% w/v rock sugar and 0.5% w/v glucose added simultaneously. After boiling at 100°C for 18 min, the mixture was added with 3% w/v jasmine green tea-leaves and soaked for 3 min. The residue was filtered out with a 120-mesh sieve. The filtrate was canned and pasteurized at 95°C for 15 min. The whole process of sample preparation strictly followed aseptic procedure, filling and sealing immediately after preparation. Prepare 6 sets of duplicate samples under the same conditions and stored hermetically at 4°C.

Preparation of FTBS: *Lactococcus lactis* subsp*. lactis* YM313 was cultured in TBS medium with 2% w/v glucose and 1.5% w/v β-sodium glycerophosphate for 2 generations, with a generation time of 15 h. *Lacticaseibacillus casei* YQ336 was cultured in TBS medium with 2% w/v glucose for 2 generations, with a generation time of 15 h. The sterilized TBS (1 kg) was fermented with a co-culture of *Lactococcus lactis* subsp*. lactis* YM313 and *Lacticaseibacillus casei* YQ336 strains (6% inoculation, YM313:YQ336 1:1), and fermented at 30°C for 15 h. Prepare 6 sets of duplicate samples under the same conditions and stored hermetically at 4°C.

### 2.2. Microbial analysis

The microbial count methods described by APHA (1984) were followed to enumerate (log CFU/ml). Dilutions were plated on MRS agar and incubated at 30°C and 48 h, followed by enumeration. Only the results of those plates that contained 30–300 colonies were considered. Each colony was microscopically inspected for strain morphology, as a way to ensure the absence of heterobacterial contamination, and the ratio of the two strains was calculated.

### 2.3. Sensory evaluation

A sensory team of 10 teachers and 5 graduate students majoring in food-related courses with sensory evaluation experience was recruited. They are healthy, non-smoking individuals with no known taste and smell defects (Perry and Hayes, 2016). The sensory evaluation was conducted in a ventilated food laboratory with sufficient light and space. All products were prepared 1 h before serving to allow for temperature and headspace equilibration. The products were labeled with different numbers, randomly disrupted, and replicated for three consecutive days under the same conditions. The samples were mainly evaluated for appearance, mouthfeel, and odor. The product appearance was evaluated by color intensity, uniformity, brightness, and gloss. Mouth feel was evaluated by richness, acceptability, and suitability for sweet and sour tastes. The odor was evaluated by the intensity of the bean odor (Perry and Hayes, 2016; Lan et al., 2020). All sensory traits were scored 1–5 (5 = excellent, 4 = good, 3 = moderate, 2 = poor, and 1 = inferior), the sum of the scores is the final sensory score of the product.

### 2.4. Electronic nose

E-nose analysis was conducted using the method (Zhu et al., 2022) with slight modifications. The flavor components of TBS and FTBS were detected by the electronic nose (PEN3, AIRSENSE Ltd., Germany). A precise 10 mL sample was placed in a 50 mL plastic tube and sealed with 3 layers of parafilm. The process parameters were as follows: injection and internal flow, 600 L/min; sample preparation time, 5 s; sample separation time, 1 s; zeroing time, 10 s; rinsing time 120 s; measuring time, 60 s. All measurements were repeated 5 times at 25°C.

### 2.5. SPME-GC–MS

SPME-GC–MS analysis was conducted using the method (Yan et al., 2022) with slight modifications. The sample (2 mL) was pipetted into a 10 mL headspace bottle and incubated in an 80°C water bath for 30 min. The solid-phase microextraction (SPME) injection needle was punctured into the headspace bottle and heating was continued for 30 min before detection by the GC–MS instrument (5975B + 6,890 N, Agilent, America). Instrument data acquisition conditions were as follows: chromatographic column (Column was stored at room temperature), HP-5MS (30 m × 0.25 mm × 0.25 μm); injection port temperature, 250°C; scan mode, Scan; ion source temperature, 230°C, splitless; quadrupole temperature, 150°C, flow velocity, 1.0 mL/min; transmission line temperature, 280°C. Temperature program: The initial temperature is set to 50°C for 2 min, the ramp rate is set to 5°C/min. The gradient 1 is set to 180°C for 5 min and ramp rate is set to 10°C/min. The gradient 2 is set to 250°C for 5 min. Qualitative analysis was performed based on the parent ion information in the full scan map followed by substance identification in the local database of Thermo NIST MS Search2.3. We matched each substance with the database three times and selected the result corresponding to the highest matching score to ensure the accuracy of the experiment. Concerning the odor, we performed an OAV (odor activity value = material concentration/aroma threshold) analysis (Perry and Hayes, 2016).

### 2.6. Analysis of pH, titratable acidity, and organic acids

The pH was determined by a pH meter (Shanghai Lei magnetic instrument Co., Ltd., Shanghai, China). Titratable acidity was titrated with 0.1 mol/L sodium hydroxide, expressed as °T. The change in contents of lactic, acetic, citric, oxalic, and tartaric acids in fermented beverages within 15 h were determined by HPLC. Two milliliter beverage was filtered through a 0.22 μm disposable needle filter for HPLC analysis under the following conditions: chromatographic column, Cosmosil C18-PAQ (250 mm × 4.6 mm, 5 μL); mobile phase, phosphoric acid: acetonitrile (pH 2.0, 98:2); detection wavelength, 210 nm. The experiments were conducted in triplicate.

### 2.7. Analysis of total flavone and phenol contents and antioxidant properties

Sample pretreatment: 5 mL sample added with 30 mL absolute alcohol was ultrasonicated for 1 h and then made 50 mL with ethanol. The mixture was centrifuged at 3,000 rpm for 10 min, and refrigerated in the dark until tested.

The total flavone content (TFC) analysis was conducted using the method (Rebollo-Hernanz et al., 2019) with slight modifications. Rutinum standard solution was added in aliquots of 1, 2, 3, 4, and 5 mL into 50 mL volumetric flask, respectively. Anhydrous ethanol was added to a total volume of 15 mL, followed by 1 mL of Al(NO_3_)_3_•9H_2_O189 solution with a concentration of 10 g/L, 1 mL of CH_3_COOK solution with a concentration of 9.8 g/L, and mixed, and then topped off to 50 mL with water. After 1 h, absorbance was measured at 420 nm, with 30% ethanol used as a blank. A standard curve was established for linear regression (y = 0.1217x − 0.0875, R2 193 = 0.9923), and the TFC content of the samples was calculated using a standard curve.

The total phenol content (TPC) analysis was conducted using the method (Sanchez Bragagnolo et al., 2022) with slight modifications. Same as the above TFC standard curve drawing method. Prepare gallic acid standard solutions of 5 concentrations for testing. Test method: 1 mL standard solution is added to 15 mL test tube, and then Folin Ciocalteu’s reagent (1 mL) and 20% Na_2_CO_3_ solution (3 mL) are added to the test tube, placed in a 50°C water bath for 30 min, and the absorbance is measured at 765 nm. (y = 0.4581x + 0.0672, *R*^2^ = 0.9965). The TPC content of the samples was calculated using a standard curve.

For the evaluation of antioxidant properties, we used the method of DPPH radical scavenging activity (Wang Y. et al., 2022), superoxide anion (O^2−^) clearance rate (Sun et al., 2018), and hydroxyl radicals scavenging rate (Wang et al., 2021) with slight modifications. The experiments were conducted in triplicate.

### 2.8. Untargeted metabolomic analysis

A total of 2 groups of samples: Bian-Que Triple-Bean Soup (TBS) and Bian-Que Triple-Bean Soup after fermentation (FTBS), each with 6 biological replicates, were analyzed. The samples were centrifuged at 3,000 × g for 10 min, and the obtained supernatants were used for analysis. Accurately 200 μL of supernatant was pipetted into a 1.5 mL centrifuge tube and added with 800 μL of extract (methanol: acetonitrile, 1:1, v:v) and 0.02 mg/mL of internal standard (l-2-chlorophenylalanine). The mixture was vortexed for 30 s and then extracted by sonication at low temperature for 30 min (5°C, 40 kHz). The samples were allowed to stand at −20°C for 30 min and then centrifuged for 15 min (13,000 × g, 4°C) to obtain the supernatant, which was dried under nitrogen. The precipitate was reconstituted in 120 μL of acetonitrile: water solution (1:1) by vortexing for 30 s and then again ultrasonicated at low temperature for 5 min (5°C, 40 kHz). The mixture was centrifuged for 10 min (13,000 × g, 4°C) and the obtained supernatant was carefully transferred to the sample vial for UHPLC–MS/MS analysis. In addition, a quality control sample (QC) was also prepared by mixing all the samples.

UHPLC–MS/MS analysis was performed on the UHPLC-QExactive HF-X system (Thermo Fisher, Inc.) using the ACQUITYUPLCHSST3 column (100 mm × 2.1 mm i.d.,1.8 μm; Waters, Milford, United States). The MS data were collected using a Thermo UHPLC -Q Exactive HF-X Mass Spectrometer equipped with an electrospray ionization (ESI) source operating in either positive or negative ion mode.

After UHPLC–MS analyses, the raw data were imported into the metabolomic processing software Progenesis QI (Waters Corporation, Milford, USA) for peak detection and alignment. At least 80% of the detected metabolic features in any set of samples were retained and normalized by sum (Liang et al., 2020). Metabolic features with a relative standard deviation (RSD) of QC > 30% were discarded. MS and MS/MS information was matched to the metabolic databases: Human metabolome database (HMDB)^1^ and Metlin database.^2^

### 2.9. Statistical analysis

Data are presented as mean ± standard deviation. The significant differences (*p* < 0.05) among different groups were evaluated by one-way analysis of variance (ANOVA) or non-parametric ANOVA. PCA analysis was performed using the inbuilt software (winmuster) of the PEN3 electronic nose, and radar plots of sensor response values were made using Excel 2010.

UHPLC–MS/MS-based metabolomic analysis: A multivariate statistical analysis (PCA, correlation analysis, OPLS-DA) was performed using ropls (Version1.6.2)^3^ in R package from Bioconductor on Majorbio Cloud Platform.^4^ Differential metabolite analysis was performed by Python packages.^5^ Statistically significant data among groups were selected with VIP value >1 and value of *p* <0.05. Differential metabolites between the two groups were summarized and subjected to Kyoto Encyclopedia of Genes and Genomes pathway enrichment analysis (KEGG).^6^

## 3. Results

### 3.1. Change in flavor and bacterial count of FTBS

**Figure 1:**
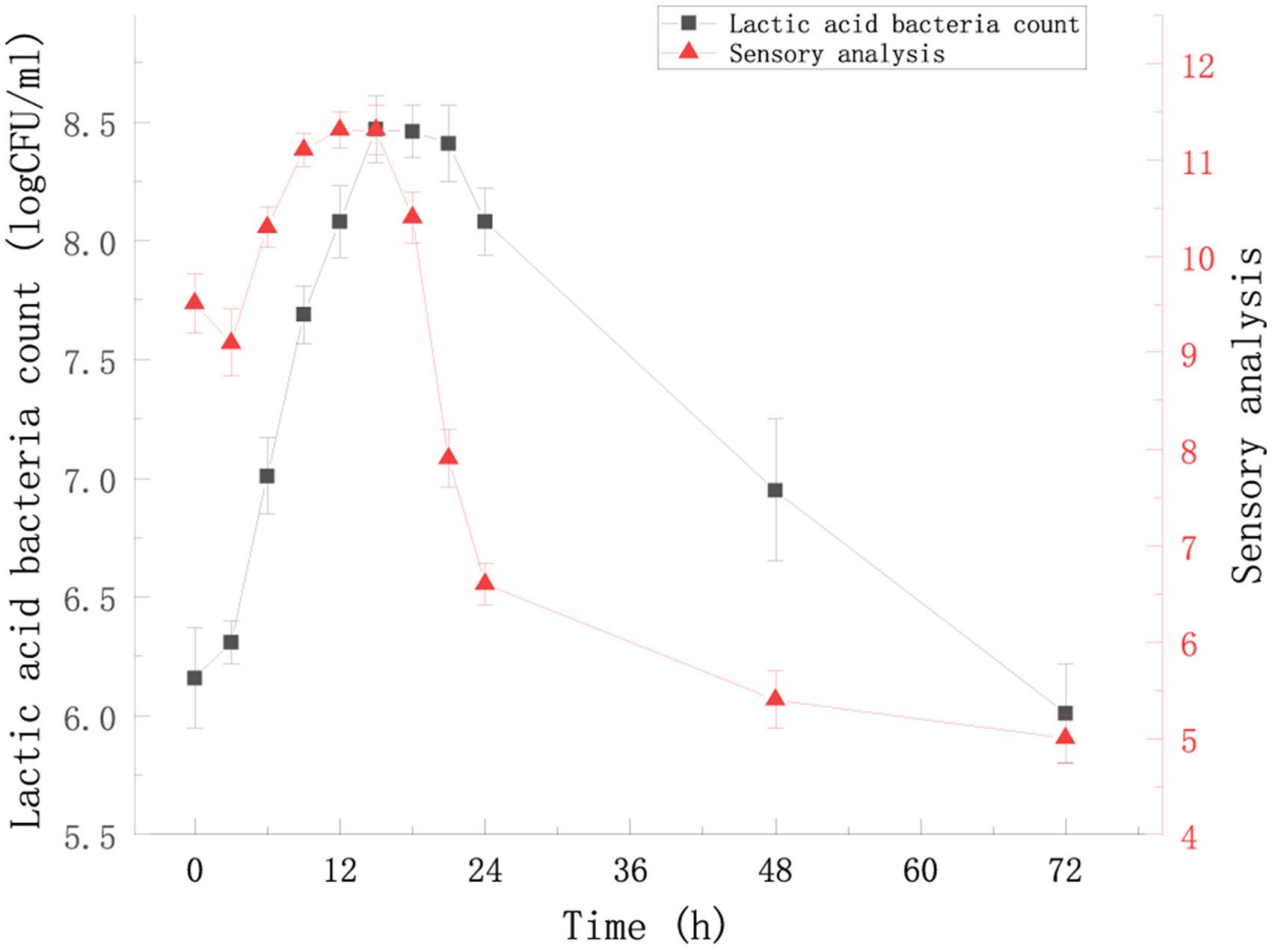
Viable counts (log CFU/mL) of YM313 and YQ336 in FTBS during a 72 h-submerged fermentation. Sensory analysis of FTBS during 72 h fermentation. Results are expressed as average (*n* = 3) ± standard deviation.

The change in the viable count and sensory score of FTBS during fermentation from 0 to 72 h were examined (Figure 1). LAB showed the fastest growth rate during 3–15 h of fermentation; the viable count peaked at 15 h to 8.47 ± 0.14 log CFU/mL. The ratio of *Lactococcus lactis* subsp*. lactis* YM313 to *Lacticaseibacillus casei* YQ336 was 1.14 ± 0.07 at this time, indicating no apparent antagonistic affects between these two strains. Figure 1A shows that during 3–15 h, the sensory scores of the samples increased with time and reached a peak at 12 h (11.31 ± 0.19) − 15 h (11.31 ± 0.26). We summarized the reasons for this phenomenon from three aspects: appearance, mouth feel and odor. With the increase in LAB, the FTBS changed color from brown to wine red, showing a uniform bright color. The sourness of FTBS gradually increased, and the ratio of sourness and sweetness gradually became appropriate. Meanwhile, LAB fermentation added a unique fermentation flavor to FTBS eliminating or masking the beany odor. This significantly improved the sensory score of FTBS. The reason for the significant decrease in sensory scores from 15 h was that LAB metabolized a large amount of sugar and produced too much organic acids, which made the sour to sweet ratio of the sample unacceptable. It is worth mentioning that the sensory team believe that the reason for the low sensory scores (9.51 ± 0.31–9.10 ± 0.35) during 0–3 h is that the mild sour taste and underfermented flavor gave the panel an impression comparable to food spoilage. In all, based on the viable count of LAB and the sensory scores, 15 h of fermentation was found optimal for FTBS.

**Figure 2:**
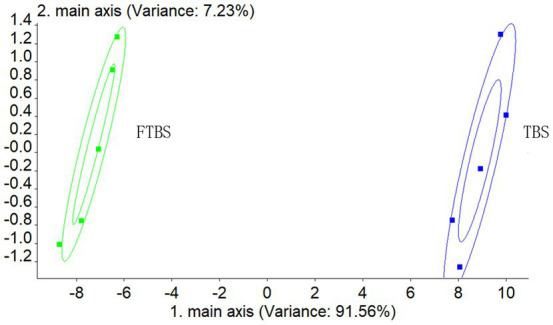
PCA analysis of electronic nose.

Next, sensory evaluation and electronic nose analysis were performed for TBS and FTBS. PCA analysis showed that the electronic nose was able to distinguish the two samples (Figure 2).

Further analysis with SPME-GC–MS distinguished the relative differences in aroma between TBS and FTBS. In total, 45 and 43 peaks were extracted in TBS and FTBS, respectively (Supplementary Figure S1). A total of 8 aroma-related compounds were extracted from these volatile substances (Table 1), of which, the percentages of methyl benzoate, nonanal, and benzyl acetate in the total volatile flavor compounds significantly decreased after fermentation. Nonanal has a strong oily odor and is one of responsible for soybean beany odor (Tang et al., 2014; Wu et al., 2018). Five new flavor compounds (acetic acid, g-terpinene, alpha-terpineol, methyl anthranilate, and damascenone) were generated in FTBS, of which, terpinene had the highest relative content. Alpha-terpineol, methyl anthranilate, and damascenone are common edible spices. Acetic acid is an important component of the typical fermented flavor of LAB fermented beverages. The increase in the proportion of these flavors and the decline of nonanal (Table 1, 2.96–2.47%) were the main reasons that improved the aroma of FTBS.

**Table 1:** Different flavor substances of TBS and FTBS.

Serial number	Retention time (min)	Name	Aroma description	Relative content (%)		Match score	
				TBS	FTBS	TBS	FTBS
1	14.472	Methyl benzoate	Floral and cherry aromas	2.10	1.48	56	64
2	14.733	Nonanal	Strong oily odor and sweet orange aroma	2.96	2.47	64	47
3	16.52	Benzyl acetate	Jasmine aroma	14.28	13.10	98	98
4	1.869	Acetic acid	Pungent sour flavor	–	1.50	–	72
5	14.586	G-terpinene	Citrus and lemon aromas	–	19.76	–	60
6	17.608	Alpha-terpineol	Syringae floral note	–	2.17	–	55
7	21.782	Methyl anthranilate	Tahara sweet aroma	–	1.52	–	80
8	22.819	Damascenone	Rose and plum, round citron, raspberry aromas	–	3.27	–	76

### 3.2. Changes in titratable acidity, pH, and organic acid content in FTBS

Organic acids (such as lactic acid, acetic acid, etc.) are common final products of LAB fermentation. Their variety and yield can affect the sensory properties of foods (Pimentel et al., 2018) and the ability of LAB to colonize and inhibit the growth of pathogenic microorganisms in the gut (Deng et al., 2020), thereby affecting the flavor and prebiotic potential of foods.

**Figure 3:**
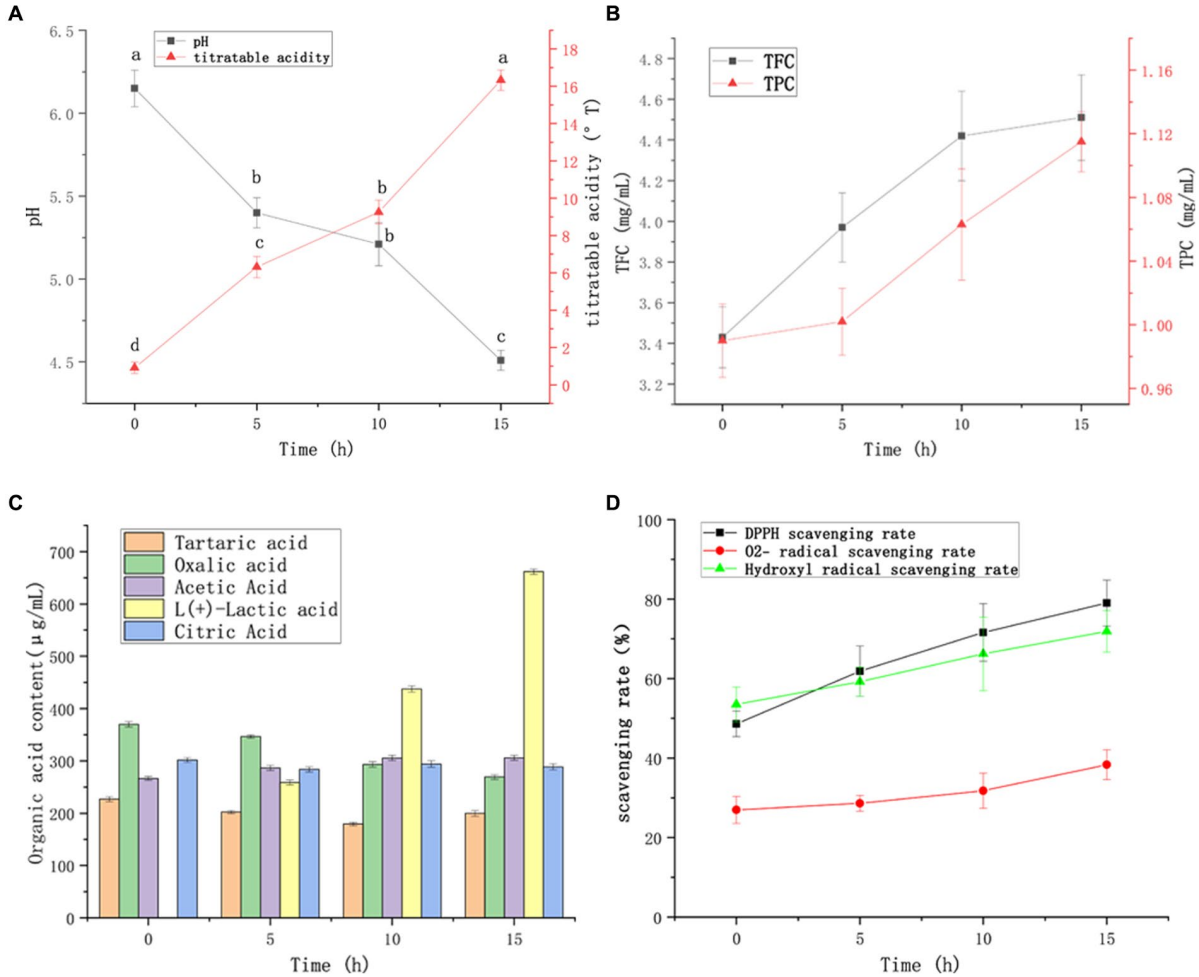
Changes in FTBS during 15 h of fermentation. Changes in the values of titratable acidity and pH **(A)**, tartaric, oxalic, acetic, lactic, and citric acids **(B)**, and TFC and TPC **(C)**. Analysis of FTBS scavenging ability against DPPH, O^2−^, and hydroxyl radicals; the results are expressed as free radical clearance **(D)**. Data are expressed as average (*n* = 3) ± standard deviation.

Changes in pH and titrated acidity during FTBS fermentation are shown in Figure 3A. During the 0–15 h fermentation process, the titrated acidity continually increased and the pH decreased. However, in the 5–10 h stage, the changes in titrated acidity and pH were smaller due to the alternating growth of *Lactococcus lactis* and *Lacticaseibacillus casei*. The pH at the initial stage of FTBS fermentation was 6.2 ± 0.1, which is optimal for the growth of *Lactococcus lactis* subsp*. Lactis* YM313 (pH, 6.6–7.2). In this period, *Lactococcus lactis* subsp*. Lactis* YM313 grew rapidly increasing the titrated acidity. During 5–10 h of the fermentation, the pH fell to 5.4–5.2, which is optimal for the growth of *Lacticaseibacillus casei* YQ336 (pH, 5.0–5.5) (Xu et al., 2019). In this period, *Lacticaseibacillus casei* YQ336 became the dominant strain that rapidly produced acid and decreased the fermentation pH (5.2–4.5).

The changes in organic acids during FTBS fermentation are shown in Figure 3B. In terms of taste, lactic acid, acetic acid, and citric acid are common souring agents in the food industry, providing typical fermented flavors for LAB fermented beverages. Among the five tested organic acids, the yield of lactic acid was the highest, followed by acetic acid. After 15 h of fermentation, lactic and acetic acid contents increased to 661.7 ± 4.9 μg/mL and 306.0 ± 4.7 μg/mL, respectively. The content of oxalic acid decreased from 370.0 ± 5.1 (before fermentation) to 269.2 ± 4.3 μg/mL.

### 3.3. Changes in bioactive substances in FTBS

TBS is rich in flavonoids and phenolic compounds, which are the most active natural antioxidants. They can scavenge free radicals, terminate free radical chain reactions, and chelate transition metals. These activities offer possible health benefits, such as reducing the risk of cancer or cardiovascular disease, and preventing or repairing cellular damage caused by reactive oxygen species (ROS). The changes in total flavone content (TFC) and total phenol content (TPC) in FTBS are shown in Figure 3C. Both TFC and TPC increased with the prolongation of fermentation time. At the end of fermentation (15 h), TFC increased to 4.51 ± 0.21 mg/mL, which was 31.5% higher than that before fermentation. Likewise, TPC increased from 0.99 ± 0.02 mg/mL before fermentation to 1.12 ± 0.02 mg/mL, showing an increase of 13.1%.

### 3.4. Changes in antioxidant activity of FTBS

The antioxidant activity of FTBS in different stages of fermentation was determined based on its free radical scavenging rates for DPPH, O^2−^, and hydroxyl radicals. The free radical scavenging rates of FTBS showed a significant increase (Figure 3D); after 15 h of fermentation, the DPPH clearance rate increased the most (62.5%) from 48.6 ± 3.2% to 79.0 ± 5.8%, the hydroxyl radical scavenging rate increased (34.4%) from 53.5 ± 4.4% to 71.9 ± 5.2%, and the O^2−^ scavenging rate increased (42.3%) from 27.0 ± 3.4% to 38.4 ± 3.7%. In all, LAB fermentation significantly improved the antioxidant activity of FTBS.

### 3.5. Metabolomics changes in small molecular substances before and after fermentation

#### 3.5.1. Metabolomics data processing and quality assessment

Continuous scans resulted in a total ion chromatogram (TIC); QC is the quality control sample, which was used to balance and stabilize the UHPLC–MS/MS system. The TIC overlap map showed a high degree of overlap and good peak separation (Supplementary Figure S2), indicating the instrument’s stability and data reliability.

**Figure 4:**
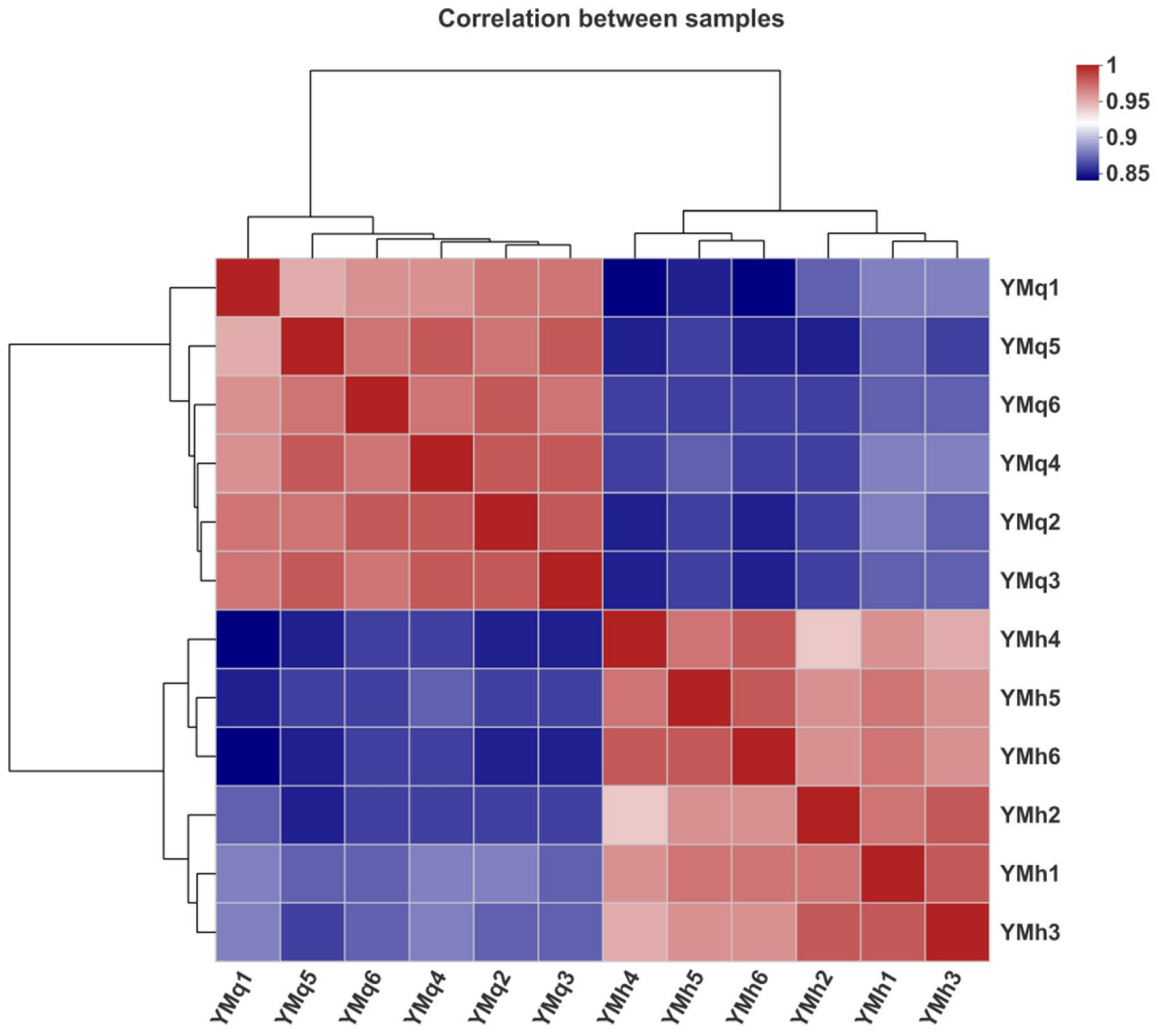
Sample correlation heat map between the 12 samples of TBS and FTBS.

Correlation analysis was performed to find differences between TBS (YMq1-6) and FTBS (YMh1-6) samples and a correlation heat map was drawn between the 12 samples (6 in each group). The parallel samples of TBS and FTBS made respective individual groups，the correlation heat map for the 12 samples showed a stronger correlation between parallel samples in the same group and indicated a good separation trend among the two groups of samples (Figure 4). This indicated that the method has good analytical stability and experimental reproducibility.

#### 3.5.2. Analysis of metabolites identified in TBS and FTBS

**Figure 5:**
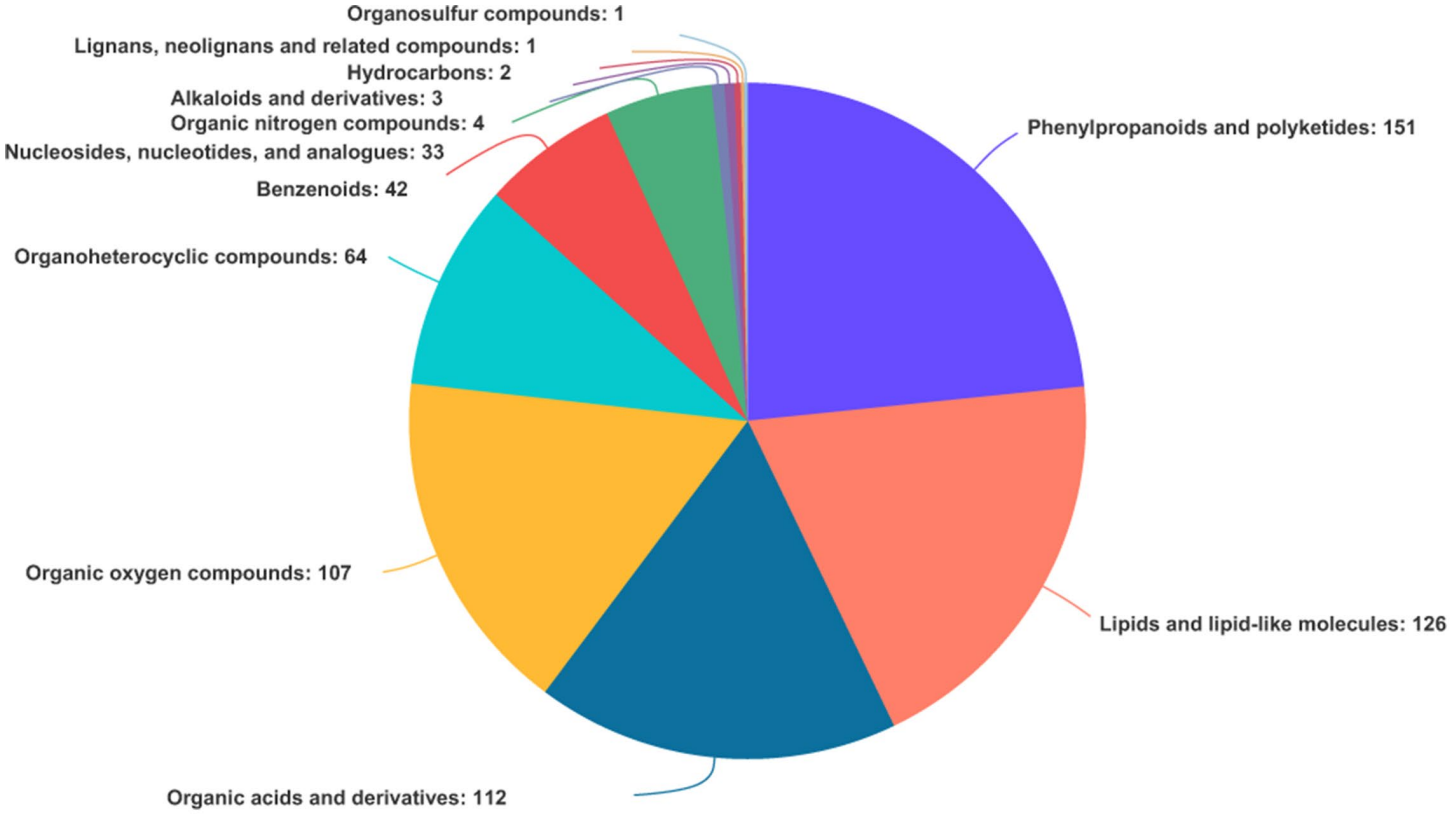
Types and proportions of metabolites identified from TBS and FTBS.

In both TBS and FTBS, a total of 646 metabolites were identified and annotated through the Human Metabolome Database (HMDB) database. The metabolites can be divided into 12 different categories (Figure 5). Among them, phenylpropane and polyketide were the most numerous with a total of 151 metabolites. They mainly included 77 flavonoid compounds [such as vitexin, apiin, (−)-epigallocatechin, etc.], 30 cinnamic acids and their derivatives (such as M-coumaric acid, sinapic acid, caffeic acid 3-sulfate, etc.), 12 isoflavones (such as glycitin, daidzein 7-O-glucuronide, 6”-O-Acetylgenistin, etc.), and 11 coumarins and their derivatives (such as 3-O-acetylepisamarcandin, aesculin, esculetin, etc.). Most of these metabolites are bioactive substances with good functional and probiotic properties. The presence of phenylpropane and polyketone compounds in TBS makes it beneficial for human immunity, diabetes, and the prevention of hand, foot, and mouth diseases.

To explore the LAB fermentation-related changes in chemical components of FTBS, the VIP values of 646 metabolites were analyzed by OPLS-DA (*p* < 0.05). Based on the fold-change (FC) of the respective metabolite, the substances with significant changes were screened out (VIP > 1 and FC < 1 or FC >1). The data obtained from the analysis and the stability of the model were checked. Both R2Y (positive 0.991, negative 0.985) and Q2 (positive 0.975, negative 0.953) scored >0.9. The intercept (Q2) was −0.2285 and − 0.2269 in positive and negative modes, respectively, which are both <0. This showed that there was no fitting phenomenon in the OPLS-DA model, indicating the feasibility of differential metabolite extraction (Supplementary Figures S3A–D).

A total of 148 differential metabolites (83 upregulated and 65 downregulated) were identified in the samples in both positive and negative modes (Supplementary Figure S4). Hierarchical clustering analysis was performed and a heat map was drawn to analyze the degree of change in the relative content of 148 differential metabolites in different samples (Supplementary Figure S5). In total, 148 differential metabolites were identified through the HMDB database. The top 30 differential metabolites were selected based on the VIP values to analyze the changes in their expression levels before and after LAB fermentation through clustering heatmaps and VIP bar charts (Supplementary Figure S6). These 30 differential metabolites include 9 organic acids and their derivatives, 6 lipids and lipid molecules, 3 organic oxygen compounds, 3 phenylpropanes and polyketones, 2 benzene ring compounds, 1 nuclear glycosides, nucleotides and analogs, 1 organic heterocyclic compound, and 5 other classes of metabolites.

Concerning the organic acids and their derivatives, the content of dipeptides (such as isoleucyl-arginine) and oxoglutaric acid (AKG) was significantly increased. AKG (FC, 1.35) is a key molecule in the TCA cycle that participates in balancing carbon and nitrogen metabolism and synthesis of phenyllactate and can delay age-related diseases (Pudlik and Lolkema, 2013; Wu et al., 2016).

In the lipid and lipid molecule category, the content of Goshonoside F5 (FC, 1.58) was significantly increased. Goshonoside F5 is known to have anti-inflammatory, antioxidant, immunity booster, and other health-benefiting properties, and can also be used as a food flavor enhancer (He et al., 2015).

Concerning the phenylpropane and polyketone, an isoflavone 3,4,5-trihydroxy-6-[4-(5-hydroxy-7-methoxy-8-methyl-4-oxo-4H-chromen-3-yl)-2-methoxyphenoxy]oxane-2-carboxylic acid (O2C, FC 1.39) and a phenylpropionic acid (phenyllactic acid, FC 1.36) were found to be significantly higher in FTBS. Isoflavones are flavonoids that are mainly found in legumes and have antiviral, antithrombotic, and antioxidant properties (Ren et al., 2018). Phenyllactic acid, produced by LAB, is a natural biological preservative with a broad antibacterial spectrum, good hydrophilicity, stability, and safety (Yang et al., 2019).

Among the organic heterocyclic compounds, ethyl maltol (FC, 3.07) was significantly increased. Maltitol (3-hydroxy-2-methyl-4-pyridone) is a naturally occurring odorless substance that can alter or enhance the flavor of foods and beverages and is marketed as a food flavor enhancer. Ethyl maltol is ~6 times more potent than maltitol (Gan et al., 2017). The significant increase in ethyl maltol content may have improved the sensory score of FTBS.

Among the other metabolites, the contents of zierin (FC, 2.65) and 5′-cytidylic acid (FC, 1.65) were significantly increased. Zierin has anti-cancer, anti-tumor, anti-inflammatory, and antioxidant properties (Tsanov and Tsanov, 2020). 5′-Cytidylic acid improves immunity and growth and has anti-oxidation, anti-inflammatory, and apoptosis lowering properties (Qian et al., 2008). Aviolin plot showing the specific changes in the levels of bioactive and flavor substances among the top 30 differential metabolites with VIP value is shown in Supplementary Figure S7.

Among the differential metabolites, apart from the top 30 metabolites, several flavonoids, isoflavones, phenolics, and other metabolites that highly correlate with antioxidant and prebiotic properties were found to be significantly increased such as 6-[3,4-dihydroxy-6-(hydroxymethyl)-5-methoxyoxan-2-yl]-5,7-dihydroxy-2-(4-hydroxy-3-methoxyphenyl)-4H-chromen-4-one, 6-[3,5-dihydroxy-2-(3-methylbut-2-en-1-yl)-4-[3-(2,4,5-trihydroxyphenyl)prop-2-enoyl]phenoxy]-3,4,5-trihydroxyoxane-2-carboxylic acid, and naringin dihydrochalcone (all three belong to flavonoids), 6”-O-acetyldaidzin (isoflavones), and 4-hydroxy-5- (4-hydroxy-3-methoxyphenyl) pentanoic acid (phenolics) (Supplementary Figure S5).

In summary, LAB fermentation effectively increased the content of bioactive substances, thus improving the functionality of FTBS. These substances have positive significance in the prevention and treatment of chronic diseases, inflammation, improvement of human immunity and antioxidation etc.

#### 3.5.3. KEGG annotation and enrichment analysis of differential metabolites

**Figure 6:**
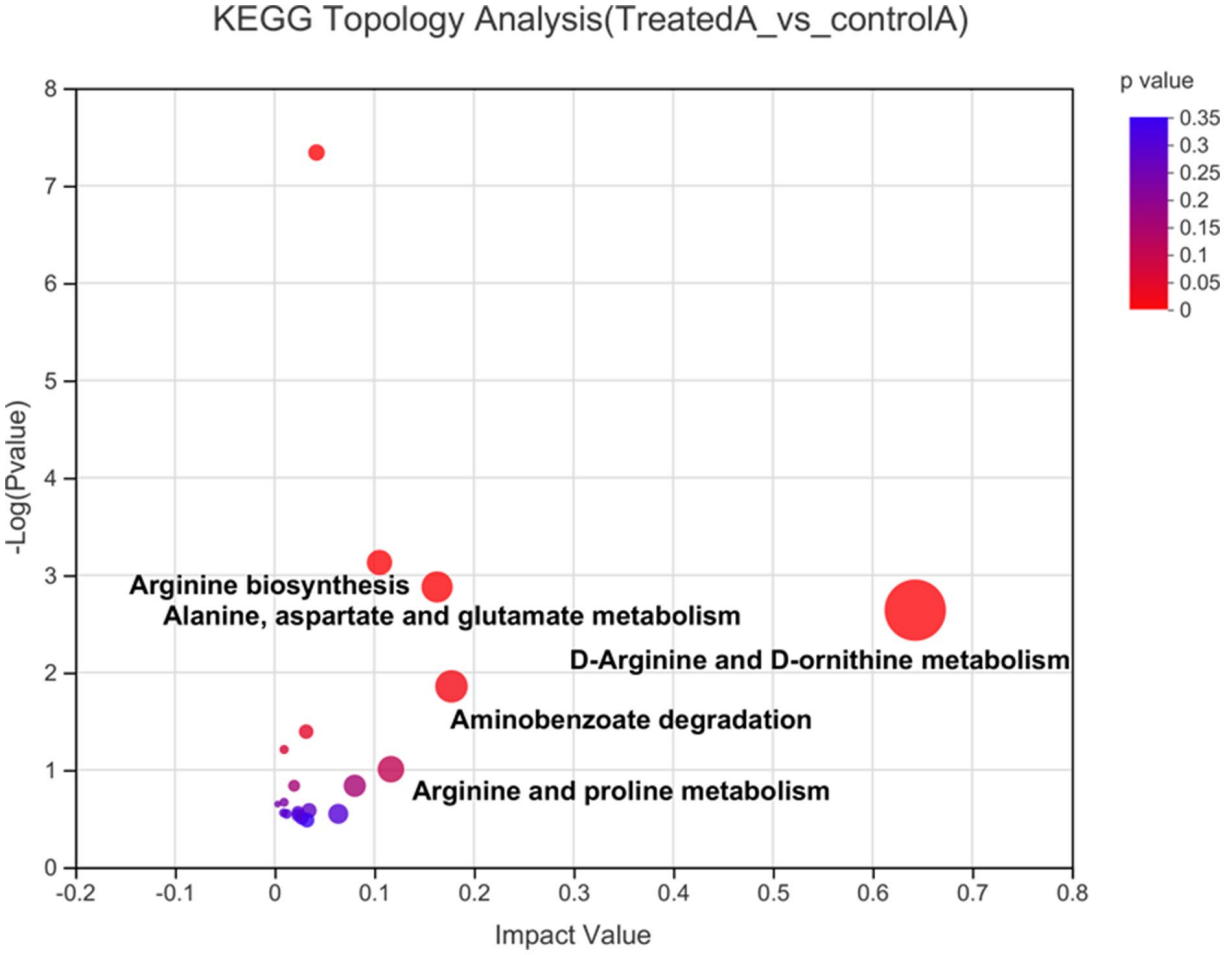
KEGG topology analysis. Each dot in the plot represents a KEGG pathway. The horizontal axis represents the impact value signifying the relative importance of metabolites in a pathway. The vertical axis indicates the enrichment significance −log10 (*p*-value) of the metabolites. Dot size represents the impact value.

KEGG pathway enrichment analysis was performed on 148 differential metabolites. In total, 58 differential metabolites, distributed in 14 metabolic pathways, were identified. Subsequently, a KEGG topology analysis was performed to identify important metabolic pathways (impact value >0.1) that included D-arginine and D-ornithine metabolism, aminobenzoate degradation, alanine, aspartate and glutamate metabolism, etc. (Figure 6). Notably, amino hydroquinone, p-salicylic acid, and 4-hydroxybenzaldehyde are the main substances of aminobenzoate degradation. LAB fermentation degrades p-salicylic acid to 4-hydroxybenzaldehyde, which has anti-thrombotic, anti-inflammatory, and antioxidant properties (Podstolski et al., 2002). The metabolites from the alanine, aspartate and glutamate metabolism pathways are L-glutamine, AKG, and L-asparagine. It may be that LAB generate AKG through this metabolic pathway.

## 4. Discussion

### 4.1. Fermentation improved sensory evaluation of FTBS

TBS has a beany odor, single mouthfeel, and a low sensory score. However, LAB fermentation improved the flavor of TBS mainly via two routes. The higher the relative concentration, the smaller the aroma threshold, and the greater the contribution of the flavor substance to the overall flavor of a food product. Among the flavors detected in TBS and FTBS, nonanal (beany odor) (Tang et al., 2014) has a low aroma threshold of 1 (Buttery et al., 1988). The proportion of nonanal decreased in FTBS, indicating that fermentation reduced the beany flavor of TBS. Acetic acid is one of the typical flavor components of LAB fermented beverages (such as Kefir) (Gamba et al., 2020), with a pungent sour odor, has a low aroma threshold. Alpha-terpineol has the aroma of the lilac flower, and g-terpinene has the aroma of citrus and lemon. Both of these are alkene compounds with a strong fragrance and low thresholds. Damascenone has a flower and fruit aroma, and methyl anthranilate has a syringae floral note and Tahara sweet aroma. The aroma thresholds are 0.002 (Buttery et al., 1990) and 8.10 (Perry and Hayes, 2016), respectively. The increase in these substances made an important contribution to the aroma of FTBS, masking the bad flavor of TBS. Blagden and Gilliland reduced the content of hexanal in soymilk through LAB fermentation (Blagden and Gilliland, 2005), but in this experiment, there was no significant change in the content of hexanal in the sample before and after fermentation, which may be due to differences in the types of beans and fermentation bacteria. Huo et al. (2023) compared the characteristics of Non-beany flavor soymilk by different LAB fermentation. They found that soymilk fermented by *Lactobacillus delbrueckii* or *Lactobacillus paracasei* had the best sensory scores. However, Huo et al. did not include *Lactococcus lactis* into the experimental object, and mixed LAB fermentation was not attempted, this experiment can be supplemented to some extent for the above experiments.

In terms of taste, the contents of lactic and acetic acids improved significantly after fermentation (Figure 3B). These are common sour agents in the food industry and provide a typical fermented flavor to LAB-fermented beverages. Among the differential metabolites, those with a significant increase are mentioned in section 3.5.3. Both goshonoside F5 and ethyl maltol are flavor enhancers and asparaginyl-proline (Supplementary Figure S5) significantly enhances the umami flavor (Zhao et al., 2021). In all, a significant increase in the content of abovesaid substances significantly improved the sensory score of FTBS.

### 4.2. Fermentation improved the functionality of FTBS

During the fermentation, the metabolic activity of LAB significantly increased the content of bioactive substances in FTBS, such as flavonoids, phenolic substances, organic acids, and other functional small molecular substances.

TFC and TPC in FTBS increased by 31.5 and 13.1%, respectively. In the plant matrix, most flavonoids and phenolics exist in the bound state, which improves their stability but reduces their biological activity. LAB fermentation improved the content and biological activity of flavonoids and phenols in FTBS by hydrolyzing the bonds between flavonoids, phenols, and the structural components of cell walls releasing more soluble active substances (Zhang et al., 2022). Flavonoids (glycosidated, methylated, and esterified) are not easily absorbed by the human gastrointestinal tract and excreted out, which reduces their bioavailability. LAB fermentation can convert bound state flavonoids and polyphenols into free aglycones improving their biological efficacy (Xiao et al., 2021; Wang Y. et al., 2022).

LAB use monosaccharides as carbon and energy source and convert them into lactic and other organic acids in FTBS. These substances can inhibit the growth of pathogenic microorganisms by reducing the pH. Figure 3B showed that *Lactococcus lactis* subsp*. Lactis* YM313 and *Lacticaseibacillus casei* YQ336 might metabolize and degrade oxalic acid, an anti-nutritional factor (Shahzad et al., 2020) in TBS. Notably, post-fermentation, the content of AKG increased significantly (Supplementary Figure S7), AKG acts as an amino receptor and facilitates the transamination reaction, which is a key factor in the phenyllactic acid biosynthesis pathway and contributes to the formation of phenyllactic acid (Pudlik and Lolkema, 2013).

The non-targeted metabolome analysis found a significant increase in the content of many bioactive small molecule substances in FTBS, such as amino acids, peptides, and their analogs (Supplementary Figure S5). Apart from the nutritional role, food proteins also promote health through biologically active peptide sequences. LAB can hydrolyze proteins by secreting proteolytic enzymes and releasing peptides into the hydrolysate (Daliri et al., 2017). Dipeptides have good functional activities. In this study, we found that fermentation significantly increased the content of several biologically active dipeptides in FTBS, such as histidylproline. Jung et al. showed that hydrolysates with high content of histidylproline exhibited high levels of free radical scavenging and oral glucose tolerance activities (Jung et al., 2011). Another increased dipeptide, arginyl-proline has an alpha-glucosidase inhibitory effect (Sartorius et al., 2019). In addition to dipeptides, the content of betaine, also known as N,N,N-trimethylglycine, was significantly increased after fermentation, which has a chemical structure similar to amino acids. It is widely regarded as an antioxidant and is used to treat liver diseases, hyperkalemia, homocystinuria, and gastrointestinal disorders (Amiraslani et al., 2012). The good antioxidant activity of FTBS may be due to the combined action of these bioactive substances, which greatly improves the prebiotic potential of FTBS.

LAB fermentation significantly improved the antioxidant activity of FTBS. This increase in antioxidant activity may come from two origins: (i) from LAB produced antioxidant metabolites such as flavonoids, polyphenols, organic acids, etc. (ii) from the LAB density that increased with the fermentation time (Figure 1). Apart from the LAB metabolic activities, LAB itself is a natural antioxidant. LAB exerts antioxidant effects by increasing antioxidant enzymes, inhibiting lipid-peroxidation reactions, scavenging free radicals, and decreasing DNA lesions (Castex et al., 2009). LAB are known to have anti-inflammatory, antioxidant, anti-cancer, and blood pressure and plasma lipid regulation activities (Liao et al., 2016). In addition, Song et al. showed that TBS can significantly reduce blood sugar levels and has excellent antioxidant capacity. Isoflavones and saponins in black soybeans and vitexin and isovitexin in mung beans are the main sources of this effect (Song et al., 2013). Vitexin, isovitexin, soyasaponin I, and soyasaponin III were also found in FTBS. The content of these substances did not significantly change after fermentation, however, the content of many other anti-diabetic-related active substances significantly increased in FTBS, such as histidylproline, arginyl-proline, and some isoflavones. Nonetheless, further experiments are needed to confirm whether LAB fermentation improves the hypoglycemic activity of FTBS.

## 5. Conclusion

The volatile flavors (8 species) and metabolites (646 species) of TBS and FTBS were comprehensively analyzed by SPME-GC–MS and non-targeted UHPLC–MS/MS. We found that LAB fermentation reduced the beany-odor of TBS and improved the overall flavor of FTBS. Also, it significantly increases the content of flavonoids, phenolic substances and other bioactive substances improving the antioxidant activity of FTBS. In conclusion, this study shows that LAB fermentation can effectively improve the overall value and promote industrial production of TBS or similar legume-based substrates.

## Data availability statement

The datasets presented in this study can be found in online repositories. The names of the repository/repositories and accession number(s) can be found in the article/Supplementary material.

## Author contributions

YL: conceptualization, methodology, software, investigation, writing—original draft, writing–review, and editing. HY: writing—review and editing. BY: resources, writing–review, and editing. JW: methodology and formal analysis. MZ: validation, resources, and data curation. JL: methodology, formal analysis, writing—original draft. ZZ: formal analysis, validation, and methodology. ZQ and LW: investigation and formal analysis. HL: resources, validation, and data curation. LZ and YX: conceptualization, supervision, and funding acquisition. All authors contributed to the article and approved the submitted version.

## Funding

This work was supported by the Liaoning Province Applied Basic Research Program (grant no. 2022JH2/101300148), Innovative Talents Support Scheme of Liaoning Higher Education Institutions (2020), 2021 Basic Scientific Research Project of Colleges and Universities of Liaoning Provincial Department of Education (grant no. LJKZ0801).

## Conflict of interest

The authors declare that the research was conducted in the absence of any commercial or financial relationships that could be construed as a potential conflict of interest.

## Publisher’s note

All claims expressed in this article are solely those of the authors and do not necessarily represent those of their affiliated organizations, or those of the publisher, the editors and the reviewers. Any product that may be evaluated in this article, or claim that may be made by its manufacturer, is not guaranteed or endorsed by the publisher.

## Footnotes

^1^http://www.hmdb.ca/

^2^https://metlin.scripps.edu/

^3^http://bioconductor.org/packages/release/bioc/html/ropls.html

^4^https://cloud.majorbio.com

^5^https://docs.scipy.org/doc/scipy/

^6^https://www.genome.jp/kegg/
