# Supplementary figures and images for: Fermentation improves flavors, bioactive substances, and antioxidant capacity of Bian-Que Triple-Bean Soup by lactic acid bacteria
Source: Front Microbiol. 2023 Jul 18;14:1152654. doi: 10.3389/fmicb.2023.1152654 (PMC10390724; doi:10.3389/fmicb.2023.1152654)

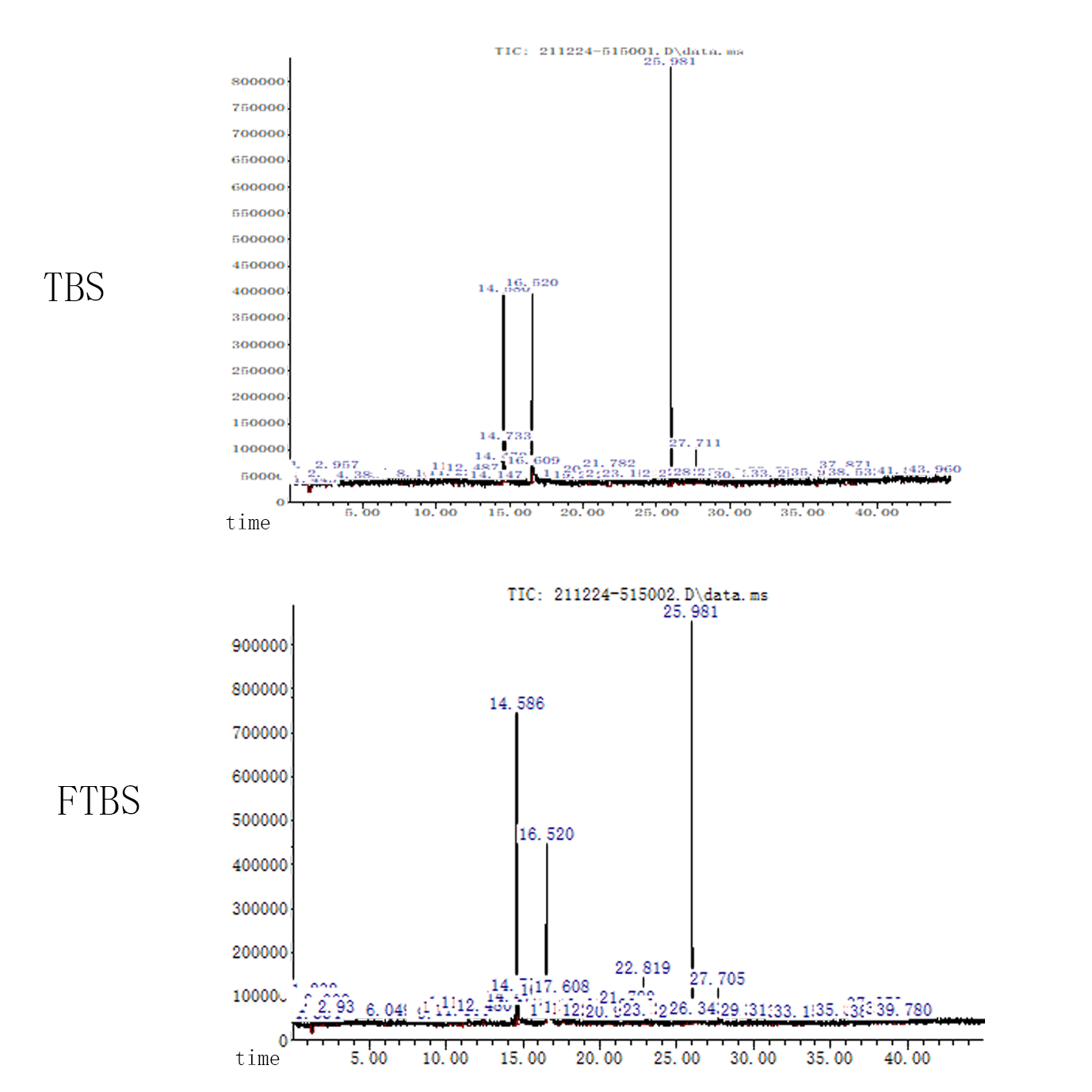

Supplement: Supplementary file 1 [file Data_Sheet_1.zip › Image 1.TIF]

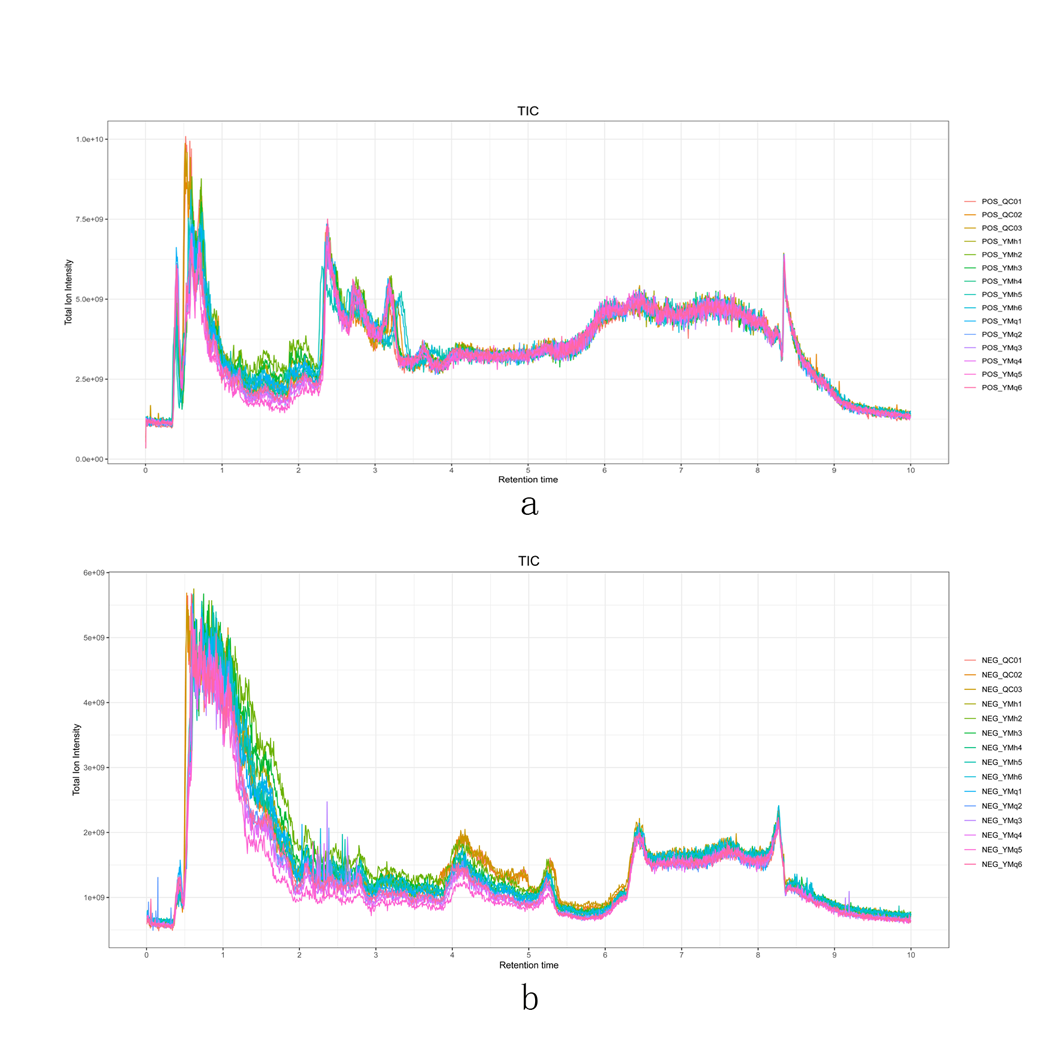

Supplement: Supplementary file 1 [file Data_Sheet_1.zip › Image 2.TIF]

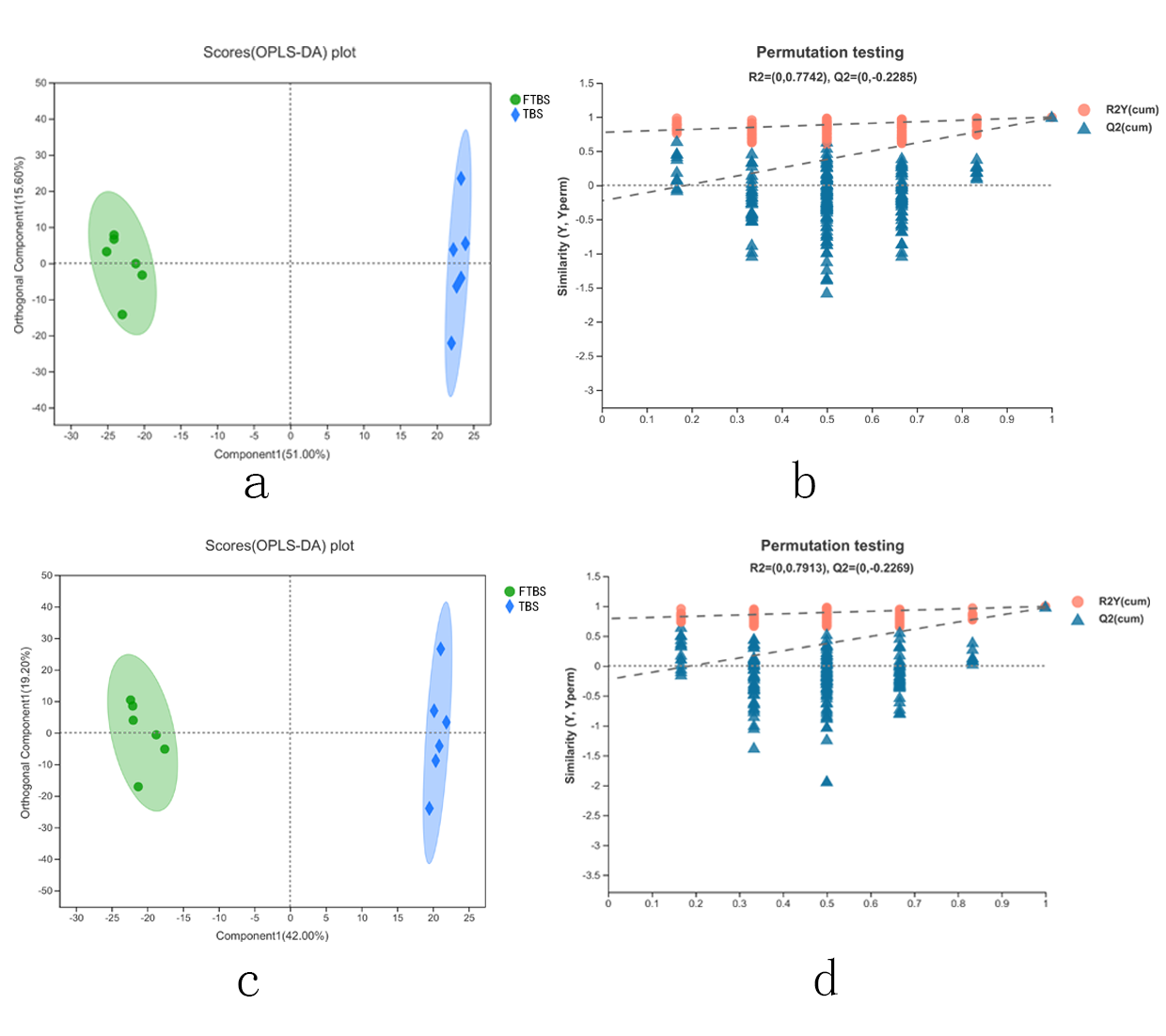

Supplement: Supplementary file 1 [file Data_Sheet_1.zip › Image 3.TIF]

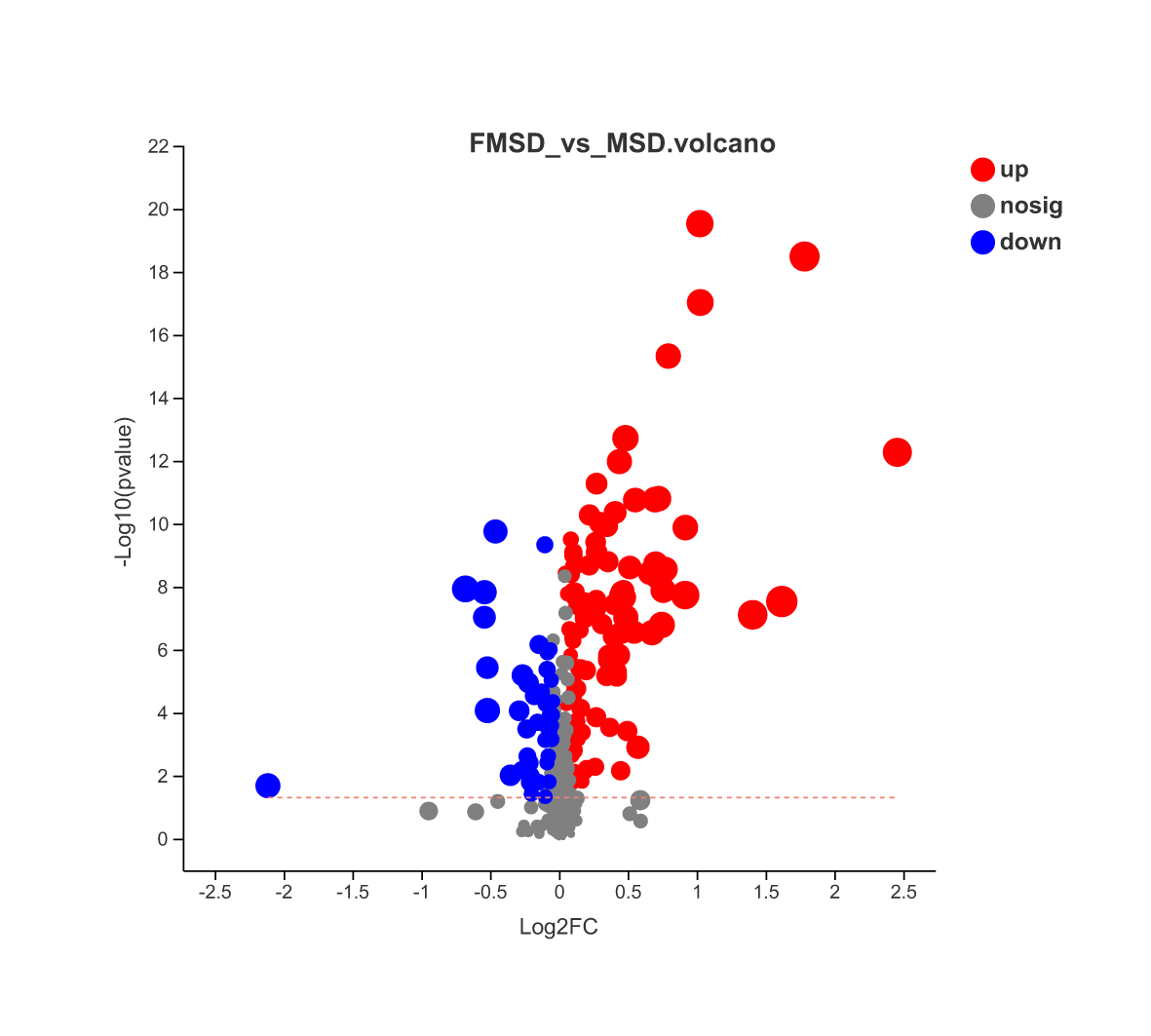

Supplement: Supplementary file 1 [file Data_Sheet_1.zip › Image 4.TIF]

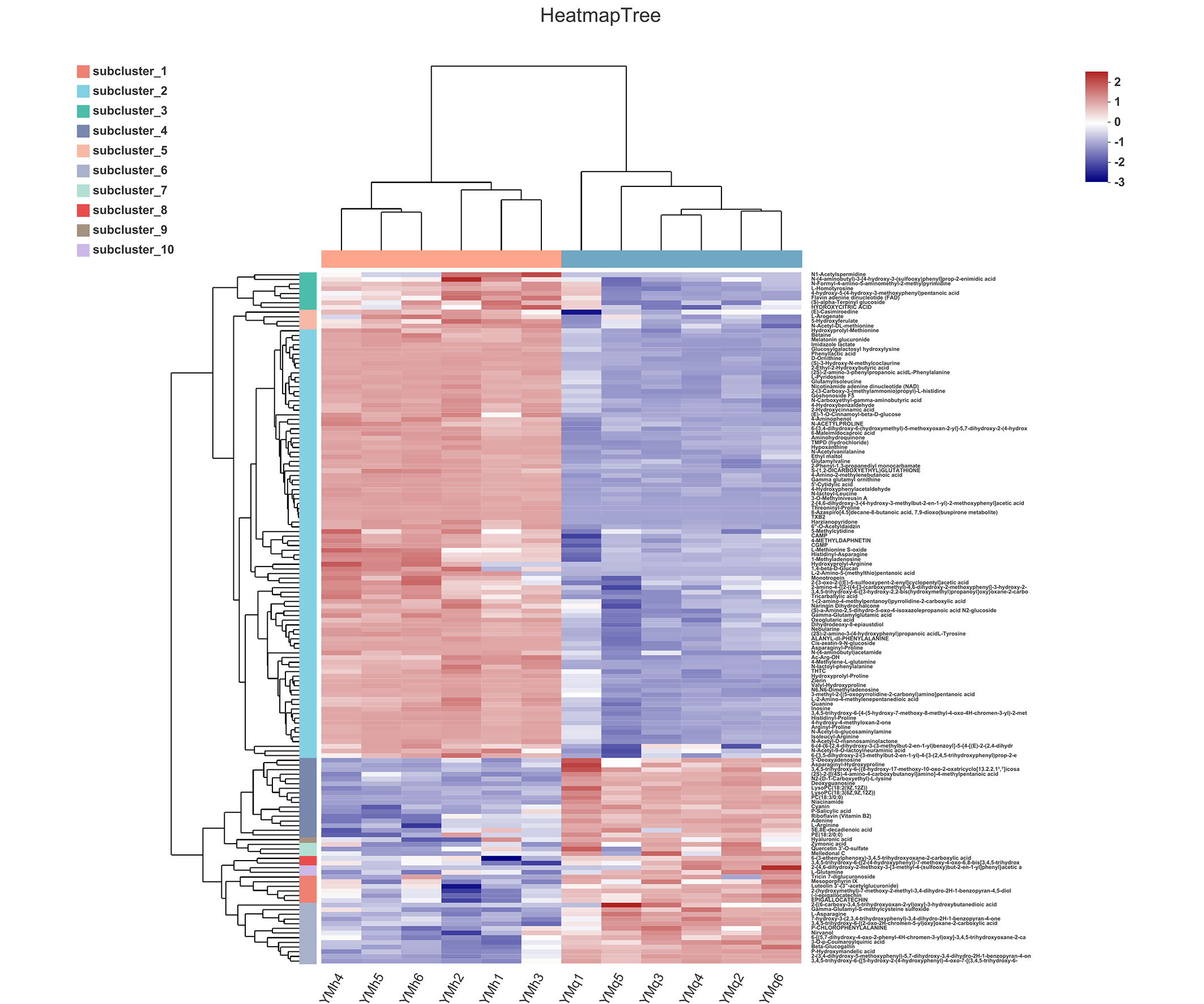

Supplement: Supplementary file 1 [file Data_Sheet_1.zip › Image 5.TIF]

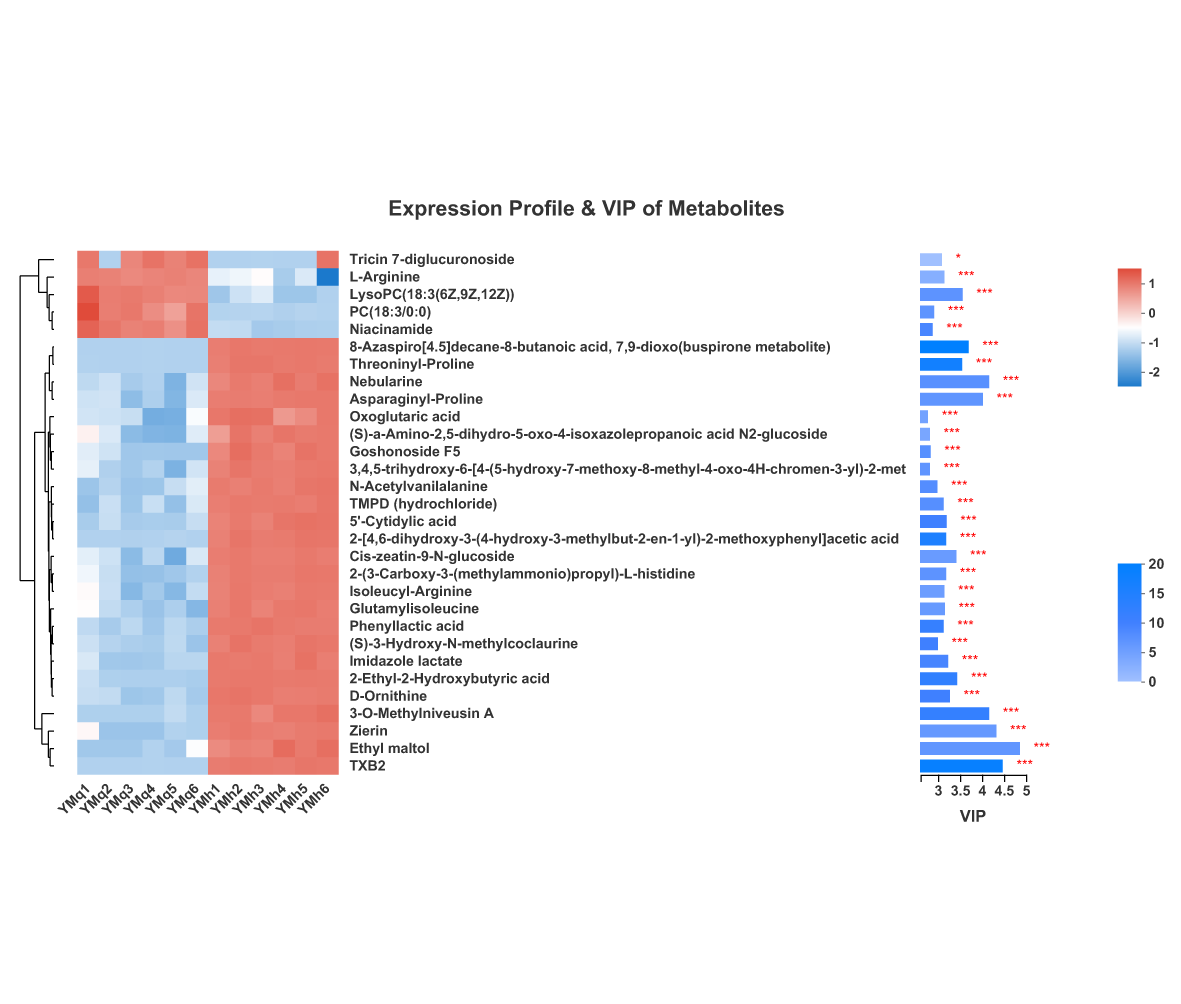

Supplement: Supplementary file 1 [file Data_Sheet_1.zip › Image 6.TIF]

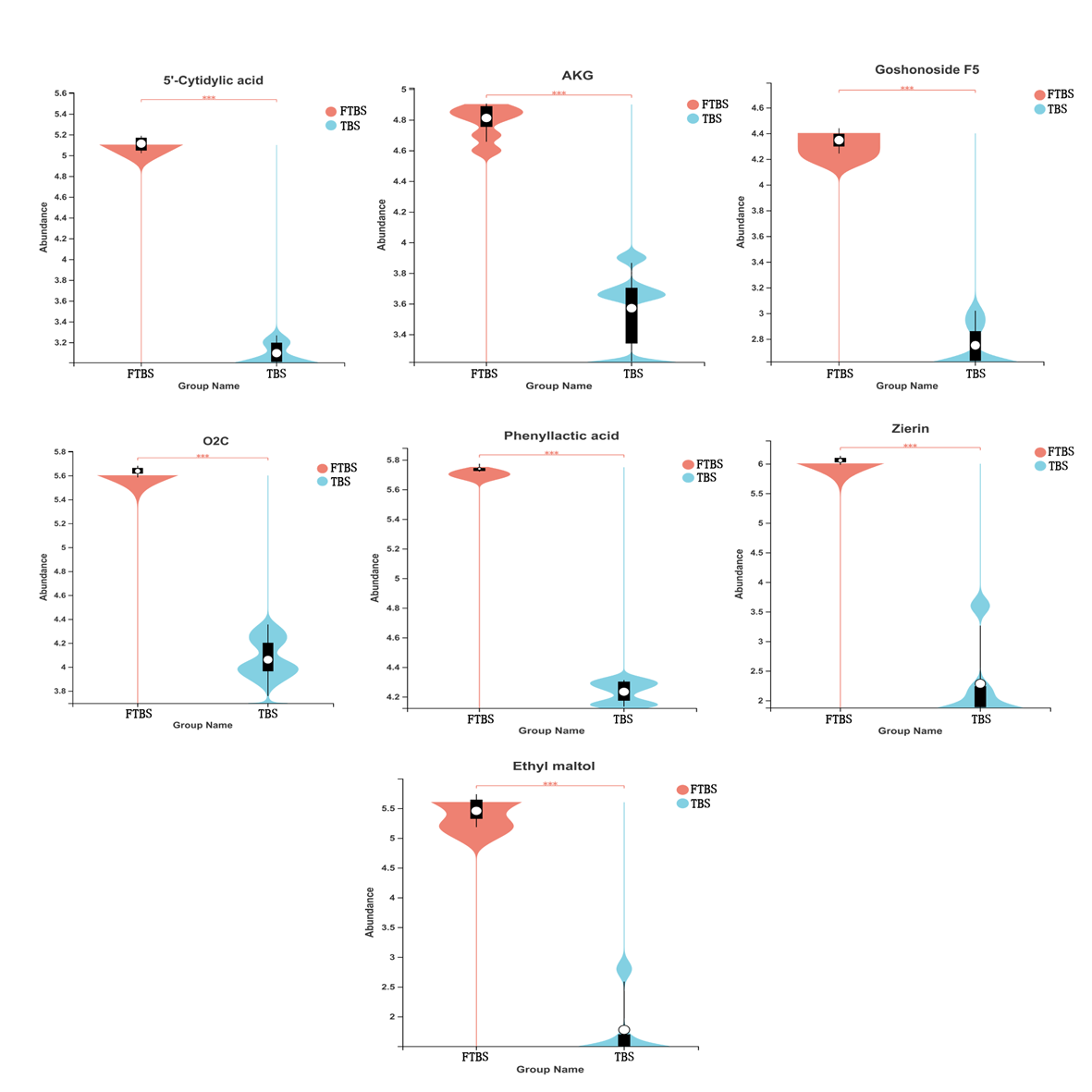

Supplement: Supplementary file 1 [file Data_Sheet_1.zip › Image 7.TIF]
